# Design, Synthesis and Antifungal Activity of Stapled Aurein1.2 Peptides

**DOI:** 10.3390/antibiotics10080956

**Published:** 2021-08-09

**Authors:** Mengjun Zheng, Ruina Wang, Si Chen, Yan Zou, Lan Yan, Linjing Zhao, Xiang Li

**Affiliations:** 1 College of Chemistry and Chemical Engineering, Shanghai University of Engineering Science, Shanghai 201620, China; 17806170387@163.com; 2 School of Pharmacy, Naval Medical University, Shanghai 200433, China; 17621069104@163.com (R.W.); zouyan@smmu.edu.cn (Y.Z.); 3 School of Medicine, Shanghai University, Shanghai 200444, China; caroline-sisi-chen@hotmail.com

**Keywords:** stapled peptide, AMP, aurein1.2, antifungal drugs

## Abstract

Aurein1.2 is a 13-residue antimicrobial peptide secreted by the Australian tree frog *Litoria aurea*. In order to improve its stabilities, the helical contents and corresponding biological activities of Aurein1.2 (a series of stapled analogues) were synthesized, and their potential antifungal activities were evaluated. Not surprisingly, the stapled Aurein1.2 peptides showed higher proteolytic stability and helicity than the linear counterpart. The minimum inhibitory concentration (MIC) of ten stapled peptides against six strains of common pathogenic fungi was determined by the microscale broth dilution method recommended by CLSI. Of them, Sau-1, Sau-2, Sau-5, and Sau-9 exhibited better inhibitory effects on the fungi than the linear peptide. These stapled Aurein1.2 peptides may serve as the leading compounds for further optimization and antifungal therapy.

## 1. Introduction

Fungal disease is an infection of human skin, mucous membranes, subcutaneous tissue, and viscera caused by fungi, which severely threatens human life and health [1,2,3,4]. *Candida* is the most common pathogen causing opportunistic fungal infection in clinic, including the invasion of the mucosa and tissues of the host and local and systemic inflammatory reactions [5]. However, the limited availability of the present clinical antifungal drugs, including a narrow antimicrobial spectrum, severe drug resistance, and obvious side effects, bring huge challenges to antifungal therapy. Therefore, the discovery of a novel antifungal agent is urgent. Furthermore, the antimicrobial peptides (AMPs) exhibit moderate advantages compared to small molecules, such as fewer side effects and low toxicity. In addition, compared with proteins, peptides have a smaller volume, which can effectively reduce the manufacturing cost [6,7].

AMPs are a class of small therapeutic peptides against microbial infections, which contain positively charged residues, like Arg or Lys, and hydrophobic residues. They possess potent activity against a wide range of Gram-negative and Gram-positive bacteria and showed low resistance compared with traditional antibiotics [8,9]. Aurein1.2 (GLFDIIKKIAESF-NH2) is an α-helical AMP consisting of 13 amino-acid residues that were secreted by the Australian tree frog *Litoria auream* [10]. Aurein1.2 is a surface-acting membrane-disrupting peptide, and the mechanism of action of Aurein 1.2 may follow the carpet mechanism. Phe3 and Phe13 may play an important role in the membrane-disrupting activity [10]. Preliminary biological studies indicated that Aurein1.2 showed moderate potency against Fungi. Accordingly, Aurein1.2 could inhibit the growth of *Candida albicans*, *Candida krusei*, *Candida tropicalis*, *Candida parapsilosis*, *Candida auris*, and *Candida glabrata* [11]. Furthermore, alanine scanning of Aurein1.2 unveiled the structure-activity relationship of Aurein 1.2 and provided insights into the furthermore optimization of Aurein 1.2 [12].

However, Aurein1.2 showed the inevitable disadvantages of normal peptides, including low bioavailability, flexible conformation, and poor membrane permeability. The poor proteolytic stability would make the peptides lose their biologically relevant conformation when crossing membranes and entering the cell [13]. Its binding selectivity was low because of the original flexible conformation [14]. Therefore, it is necessary to constrain the helical conformation, enhance the proteolytic stability, and thus, increase the biological activities of Aurein1.2.

In recent years, an all-hydrocarbon peptide stapling strategy was developed by Verdine et al. and shown to be capable of reinforcing the helicity and stability of peptides and has been well used in peptide drug design [15,16,17,18,19]. In this study, to improve the performance of Aurein1.2, we designed a series of stapled Aurein1.2 peptides by using the all-hydrocarbon peptide stapling strategy, which can help Aurein1.2 improve its helical contents, proteolytic stability, and more importantly, antifungal properties.

## 2. Results

### 2.1. Stapled Peptides Design and Synthesis

**Figure 1:**
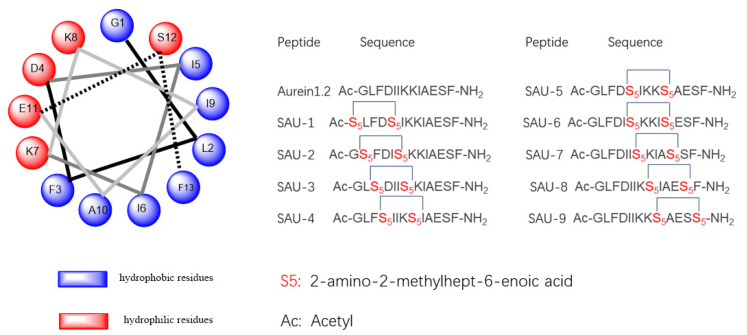
The sequences of Aurein1.2 and the stapled Aurein1,2 peptides.

**Figure 2:**
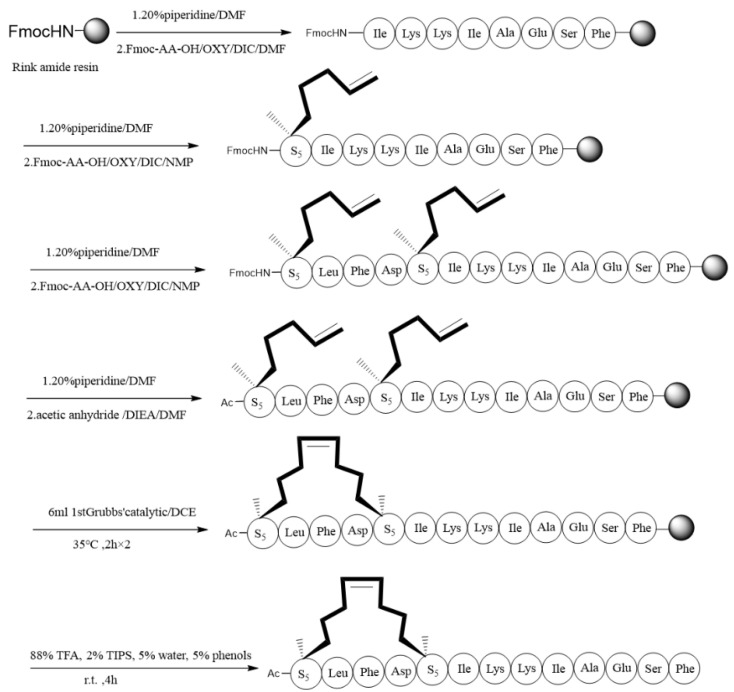
The synthetic route of stapled peptide Sau-1.

We designed the hydrocarbon (stapled) analogues of Aurein1.2 to explore the influence on the stability, helicity, and antifungal activities of Aurein1.2 using all-hydrocarbon peptide stapling. Additionally, we choose i, with i + 4 as the stapling position and one-helix-space [20]. Accordingly, a series of stapled peptide analogues of Aurein1.2 were designed (Figure 1). Using conventional solid-phase peptide synthesis (SPPS) methods, we obtained a crude linear peptide precursor with a two-terminal olefin anchored residue [21]. Rink amide resin was used for solid-phase support, and the amine was protected with Fmoc (Figure 2) [22]. The assembled linear peptides, under the action of Grubb’s first-generation catalyst, cyclized the peptide chain. Then peptides were cleaved from resin to get crude peptides. Crude peptides were purified by RP-HPLC. Finally, they were identified with high-performance liquid chromatography (HPLC) and high-resolution mass spectrometry (HR-MS) [23] (Supplementary Material).

### 2.2. Helicity Degree

**Figure 3:**
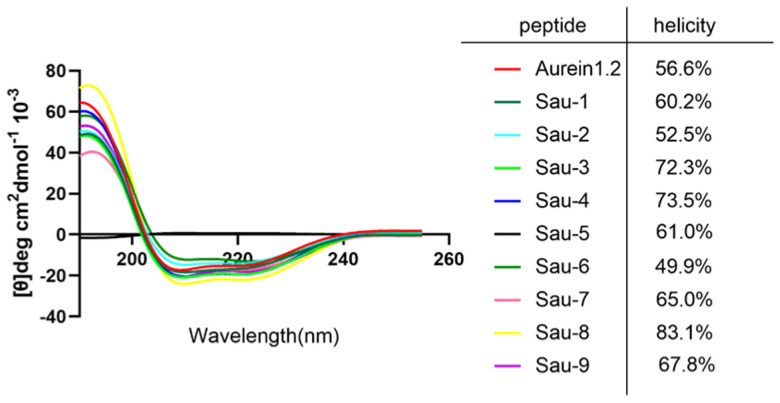
CD spectra of Aurein1.2 and its analogues. The peptides were dissolved in a solution of phosphate buffer and trifluoroethanol (7:3) and reached a concentration of 50 μM. The helicities of these peptides were calculated on the [θ]_222_.

We use circular dichroism (CD) spectroscopy to probe the structural changes of Aurein1.2. According to the formula for calculating the helix reported in the previous relevant literature, we calculated the helicities of Aurein1.2 and the stapled peptides [24]. As Figure 3 shows, the helicities of Aurein1.2 and the stapled peptides were measured. The CD data indicated that the helicity of Aurein1.2 was 56.6%, and the helicities of the stapled peptides were 60.2%, 52.5%, 72.3%,73.5%, 61.0%, 49.9%, 65.0%, 83.1% and 67.8% for Sau-1, Sau-2, Sau-3, Sau-4, Sau-5, Sau-6, Sau-7, Sau-8 and Sau-9, respectively. These results indicate that the helical contents of most stapling peptides are higher than that of the linear peptide; that is, all-hydrocarbon stapling could enhance the helical contents of stapled analogues.

### 2.3. Protease Stability Analysis

**Figure 4:**
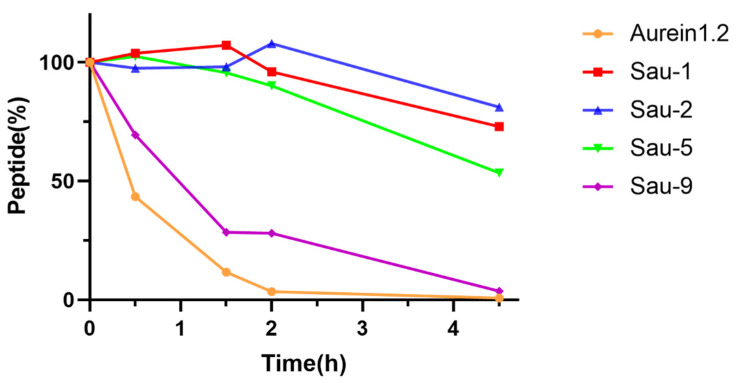
Proteolytic stability of Aurein1.2, Sau-1, Sau-2, Sau-5, and Sau-9. They were dissolved in chymotrypsin solution (10 ng/μL, 50 mM, containing 2 mM of CaCl_2_, pH = 7.4). The percentages of the remaining peptides were monitored by HPLC.

Sau-1, Sau-2, Sau-5, and Sau-9 with relatively good activities were selected to study the proteolytic stability of the stapled peptides and compared with Aurein1.2. Their susceptibility toward chymotrypsin degradation was tested in pH 7.4 PBS buffer at room temperature, containing 2 mM of CaCl_2_, and the percentages of remaining peptides were determined by HPLC. The C-terminal of the peptide hydrolytic site of chymotrypsin contains mainly aromatic amino acids, including phenylalanine (Phe), tryptophan (Trp), and lysine (Tyr), and has also been reported to hydrolyze Leu. As Figure 4 shows, Aurein1.2 was disintegrated almost completely by 4.5 h. Interestingly, under the same conditions, the data demonstrated that both Sau-1, Sau-2, and Sau-5 remain relatively stable over time. Sau-9 was observed to be less stable than the other three stapled peptides and was slightly more stable than Aurein1.2. The results showed that most of the stapled peptides were more stable than the linear peptide. The corresponding HPLC charts for the determination of peptide residues are in the Supplementary Material.

### 2.4. Determination of MIC for Candida Species

The in vitro antifungal activities of the target peptides were evaluated according to protocols from the CLSI. The broth microdilution method was used to determine the minimum inhibitory concentration (MIC) of the target peptides in 96-well plates. Fluconazole was used as a reference drug. As shown in Table 1, Sau-1 inhibited the growth and reproduction of fluconazole-resistant clinical isolates of *C. albicans* 901 (MIC 32 μg/mL) and *C. parapsilosis* ATCC22010 (MIC 128 μg/mL). Sau-2 inhibited the fluconazole-resistant isolate *C. tropicalis* 895 with MIC of 16 μg/mL. Sau-5 inhibited *C. albicans* SC5314 and *C. tropicalis* 895, both with MIC of 16 µg/mL. Sau-9 can inhibit *C. tropicalis* 895 with an MIC of 32 µg/mL. Our results indicated that the stapled peptides possessed antifungal activities. Moreover, the antifungal activities of some stapled Aurein1.2 peptides were higher than that of the linear peptide.

## 3. Discussion

In general, peptides can be modified by the methods of lipidation, glycosylation, and cyclization to enhance their protease resistance and improve their antibacterial activities [25]. In this case, we designed and synthesized a series of stapled Aurein1.2 peptides that exhibited improved proteolytic stability and higher helical contents than their linear counterparts. Importantly, the antifungal experiment indicated that these stapled peptides could effectively enhance the potency of Aurein1.2 towards some *Candida* species so as to provide the basis for the development of antifungal drugs in the future. Compared with Aurein1.2, Sau-1, Sau-2, Sau-5, and Sau-9 have slightly enhanced effects against some *Candida* strains. Specifically, Sau-1 has inhibitory effects against the fluconazole-resistant *C. albicans* isolate 901. Sau-2, Sau-5, and Sau-9 have higher effects than the fluconazole against the *C. tropicalis* isolate 895. Overall, these stapled Aurein1.2 peptides may serve as the leading compounds for the future development of antifungal drugs.

It is not surprising that the antifungal activities of the stapled peptides are better than that of the linear peptide, and the helicity may be related to the antifungal activities. The potential correlation between the helical contents of the peptides and the biological activities has previously been shown [26], and a positive correlation does exist in this case. Therefore, it can be reasonably explained that the antifungal activities of stapled peptides were significantly improved compared with that of the linear peptide. Furthermore, a possible risk of Aurein1.2 peptide is its instability to enzymes, which could be improved through hydrocarbon stapling [26]. Sau-1, Sau-2, and Sau-5 obviously better resist the chymotrypsin hydrolysis than the linear counterpart. We speculate that the improved helical structures of stapled peptides reduce the leakage at the enzymatic hydrolysis site.

In summary, we report the first synthesis of a series of stapled peptides based on the Aurein1.2 sequence. The stapled peptides show higher proteolytic stability and helical contents (so as to enhance antifungal activities) relative to the linear peptide. Therefore, stapled Aurein1.2 peptides may be leading compounds for further optimization and antifungal therapy.

## 4. Materials and Methods

### 4.1. Materials

We use the Rink amide resin, bought in Nankai Hecheng Science & Technology Co. Ltd. (Tianjin, China). We use all reagents and solvents which were purchased from Chemical Reagent Co. Ltd. (Shanghai, China), Qir biochem, or Titan Scientific Co. Ltd. (Shanghai, China), without further purification.

### 4.2. HPLC

The Waters and Agilent HPLC (high-performance liquid chromatography) systems were used for HPLC analysis and preparation. The analytical HPLC equipment used an analytical column (XB ridge C18, 5 μm particle size, 150 mm × 4.6 mm, flow rate 1.0 mL/min, 30 °C). The preparative column (YMC-Pack ODS-AQ C18, 10 μm particle size, 250 × 20 mm, flow rate 30 mL/min) was also used; both used the same mobile phase system, which was composed of solution A (0.1% trifluoroacetic acid in water) and solution B (0.1% trifluoroacetic acid in acetonitrile). They were monitored at 214 nm, and 254 nm and the column temperature was 30 °C. For the analytical HPLC, the gradient followed: 0–5 min, 5–5% B; 5–30 min, 5–65% B; B. For the preparative HPLC, the gradient followed: 0–5 min, 25–25% B; 5–60 min, 25–45% B; 60–80 min, 80–80% B.

### 4.3. Stapled Peptides Synthesis

We utilized the conventional solid-phase peptide synthesis (SPPS). Firstly, 333 mg Rink Amide resin was soaked in dichloromethane (DCM) for 20 min at room temperature to induce swelling. Then, the Rink Amide resin was treated with Oxyme/piperidine/DMF (71:2:4 = m/v/v 5 min × 2) for the deprotection. Afterwards, we washed the resin with DMF, DCM, and DMF five times, five times and two times, respectively. To ensure the amino acid link to the resin, we added 0.5 mmol Fmoc-AA-OH, 0.5 mmol OXY, 0.5 mmol DIC, and about 7 mL NMP to the resin. Then, the mixture was placed on a shaking table at 60 °C. It needed 20 min to connect with the resin, followed by washing with DMF (5 times), DCM (5 times), and DMF (2 times). Each amino acid was detected with phenol and ninhydrin until there was no colour reaction. It started deprotection, followed by washing with DMF (5 times), DCM (5 times), and DMF (2 times). After deprotection, the next amino acid could be connected. All steps, including deprotecting, washing, coupling, and re-washing were needed to be repeated until all amino acid residues were linked to the resin. For particular amino acids, S_5_ (2-amino-2-methylhept-6-enoic acid), we need to add 1.5 equiv. of the Fmoc-protected amino acid, and the required reaction time was 2 h. Moreover, the amino acid located behind the S_5_ also needed 2 h to connect with the peptide. When the last amino acid was linked to the resin, DMF, DCM, and DMF were used to wash the resin five times, five times, and two times, respectively. Then the mixture of Oxyme, piperidine, and DMF (71:2:4 = m/v/v) was used to remove the Fmoc group from the N-terminus, followed by washing with DMF (five times), DCM (five times) and DMF (two times). The mixture of DIEA, acetic anhydride and DMF was added to the resin at room temperature for 20 min to react on an oscillating table. In the presence of Grubbs’ first-generation catalyst and 1,2-dichloroethane (DCE) solvent, the assembled linear peptide cyclized the peptide chain. The solution of Grubbs’ first-generation catalyst was dissolved in DCE, and the solution was added to the resin to react for 2 h. After the first 2 h, a fresh solution of Grubbs’ first-generation catalyst was added to the resin for another 2 h. The resin was washed with DMF (5 times), DCM (5 times) and DMF (2 times). The cocktail B regent (88% TFA, 2% TIPS, 5% water, 5% phenols) was added to the resin at room temperature for 4 h to make the cleavage of the peptide from the resin. Then ice ether was added to the solution, and the mixture was centrifuged at 3600 r/min for 3 min. Then the supernatant was poured out, fresh diethyl ether was added and centrifuged again. This was repeated 3–4 times, then the supernatant was poured out, and the sediment was dried in nitrogen to get crude peptides.

### 4.4. Circular Dichroism

The linear peptide and the stapled Aurein1.2 peptides were dissolved to form a sample with a concentration of 50 μM. The solvent consists of phosphate-buffered solution and trifluoroethanol (7:3). We used a 1 nm quartz tube to obtain CD spectra on a Jasco-715 spectrophotometer at 20 °C. The measurement parameters were set up as follows: wavelength, 190–255 nm; speed, 20 nm min^−1^; step resolution, 1.0 nm. And we accumulated the data twice. All spectral data were subtracted from the background. The curves were smoothed with standard parameters. The helicity of each peptide was calculated according to the equation in the literature [27].

### 4.5. Chymotrypsin Digestion Assay

Aurein1.2, Sau-1, Sau-2, Sau-5, and Sau-9, were dissolved in dimethyl sulfoxide to prepare a reserve solution with a concentration of 1 mM. Chymotrypsin was dissolved in PBS buffer (50 mM, containing 2 mM of CaCl_2_, pH = 7.4) to obtain a final concentration of 10 ng/µL. Then the 130 µL peptide solutions were incubated with 910 µL of chymotrypsin solution at room temperature. Then 130 µL of digestion mixture was taken at 0, 0.5, 1.5, 2 or 4.5 h, and then quenched with 50 µL of hydrochloric acid (1 mM). High-performance liquid chromatography (HPLC) was used to detect the residues of linear peptides and stapled Aurein1.2 peptides at 214 nm at a different time.

### 4.6. Strains and Medium

Clinical isolates of *C. albicans* SC5314, 901, 904, *C. tropicalis* ATCC20026 and clinical isolate 895, *C. glabrata* ATCC1182 and clinical isolate 896, *C. krusei* ATCC2340, and *C. parapsilosis* ATCC22010 are from the collection library in the Drug Discovery and Development Center, Naval Medical University, Shanghai, China. *C. auris* clinical isolates are provided by Linqi Wang (Institute of Microbiology, Chinese Academy of Sciences) and Changbin Chen (Institut Pasteur of Shanghai, Chinese Academy of Sciences). All strains were cultured in YPD (1% yeast extract, 2% peptone, and 2% glucose) liquid medium overnight at 30 °C with shaking at 220 rpm.

### 4.7. Determination of MIC for Candida Species

The MICs of chemicals against all strains were determined by the micro broth dilution method according to the Clinical and Laboratory Standards Institute (Wayne PA, Reference Method for Broth Dilution Antifungal Susceptibility Testing of yeasts, approved Standard M27-A3, 3 rd ed; Clinical and Laboratory Standards Institute (CLSI): USA, 2008). The initial concentration of fungal suspension in RPMI 1640 medium was 3–5 × 10^3^ colonies forming unit (CFU)/mL, which was added to 96-well plates. Peptides were prepared in dimethyl sulfoxide (DMSO) with an initial stored concentration of 6.4 mg/mL. Serial dilutions were made based on the CLSI M27 guide to obtaining the final concentrations ranging from 0.125 to 64 μg/mL for fluconazole and 0.25 to 128 μg/mL for peptides and fluconazole. The plates were incubated at 30 °C for 48 h. Optical density was measured at 630 nm, and background optical densities were subtracted from that of each well. The definition of MIC is the lowest concentration at which 80% of tested strains cannot grow.

## Figures and Tables

**Table 1:** Minimum inhibitory concentration (MIC, μg/mL) of the target peptides against *Candida* isolates.

Peptide	*C. albicans*			*C. tropicalis*		*C. glabrata*		*C. auris*		*C. krusei*	*C. parapsilosis*
	SC5314	901	904	ATCC 20026	895	ATCC 1182	896	918	919	ATCC 2340	ATCC 22010
Sau-1	>64	32	>128	>128	>128	>128	>128	>128	>128	>128	128
Sau-2	>64	>128	>128	>128	16	>128	128	>128	>128	>128	>128
Sau-3	>64	>128	>128	>128	>128	>128	>128	>128	>128	>128	>128
Sau-4	>64	>128	>128	>128	>128	>128	>128	>128	>128	>128	>128
Sau-5	16	>128	>128	>128	16	>128	>128	>128	>128	>128	>128
Sau-6	>64	>128	>128	>128	>128	>128	>128	>128	>128	>128	>128
Sau-7	>64	>128	>128	>128	>128	>128	>128	>128	>128	>128	>128
Sau-8	>64	>128	>128	>128	>128	>128	>128	>128	>128	>128	>128
Sau-9	>64	>128	>128	>128	32	>128	>128	>128	>128	>128	>128
Aurein1.2	>64	>128	>128	>128	>128	>128	>128	>128	>128	>128	>128
Fluconazole	0.5	>64	>64	1	>128	2	>128	>128	>128	64	1

## Data Availability

Data is contained within the article or Supplementary material.

## Supplementary Materials

The following are available online at https://www.mdpi.com/article/10.3390/antibiotics10080956/s1, Figures S1–S10: HR-MS for compound Aurein1.2, SAU-1, SAU-2, SAU-3, SAU-4, SAU-5, SAU-6, SAU-7, SAU-8, and SAU-9. Figures S11–S20: Analytical HPLC trace for compound Aurein1.2, SAU-1, SAU-2, SAU-3, SAU-4, SAU-5, SAU-6, SAU-7, SAU-8, and SAU-9. Figures S21–S24: Analytical HPLC trace for the residual content of compound Aurein1.2, SAU-1, SAU-2, SAU-5, SAU-9. Figures S25–S28: The hydrolytic stability of Aurein1.2 and SAU -1/SAU-2/SAU-5/SAU-9 was determined by high-performance liquid chromatography. Table S1: Electrospray MS data for peptides (positive mode).
