# Supplementary material for: Design, Synthesis and Antifungal Activity of Stapled Aurein1.2 Peptides
Source: Antibiotics (Basel). 2021 Aug 9;10(8):956. doi: 10.3390/antibiotics10080956 (PMC8389037; doi:10.3390/antibiotics10080956)
Supplement: Supplementary file 1 [file antibiotics-10-00956-s001.zip › antibiotics-1310705-supplementary.pdf]

Supplementary Material

Table S1. Electrospray MS data for peptides (positive mode)

Peptide	Molecular Weight (g/mol)	Found Mass
Aurein1.2	1520.86	$[M+H]^+$ = 1521.8632;
Sau-1	1600.96	$[M+H]^+$ = 1601.9678;
Sau-2	1544.86	$[M+H]^+$ = 1545.8645;
Sau-3	1495.87	$[M+H]^+$ = 1496.8702;
Sau-4	1527.90	$[M+H]^+$ = 1528.9080;
Sau-5	1544.86	$[M+H]^+$ = 1545.8657;
Sau-6	1586.91	$[M+H]^+$ = 1587.9116;
Sau-7	1513.89	$[M+H]^+$ = 1514.8955;
Sau-8	1555.90	$[M+H]^+$ = 1556.9061;
Sau-9	1510.88	$[M+H]^+$ = 1511.8856;

Spectrum

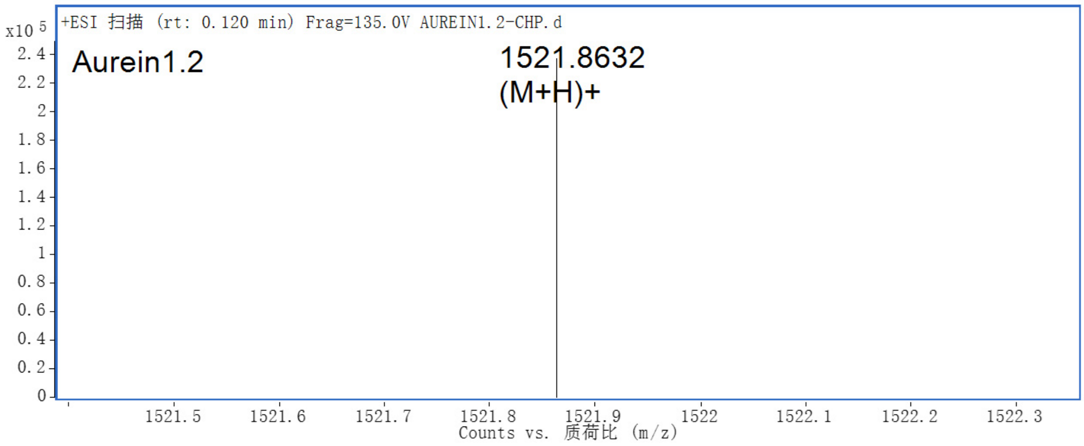

Figure S1. HR-MS for compound **Aurein1.2**

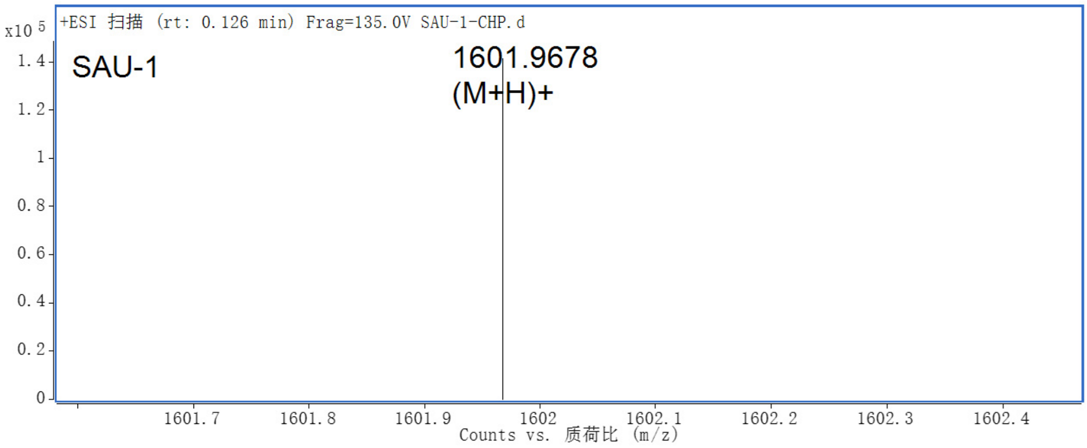

Figure S2. HR-MS for compound **SAU-1**

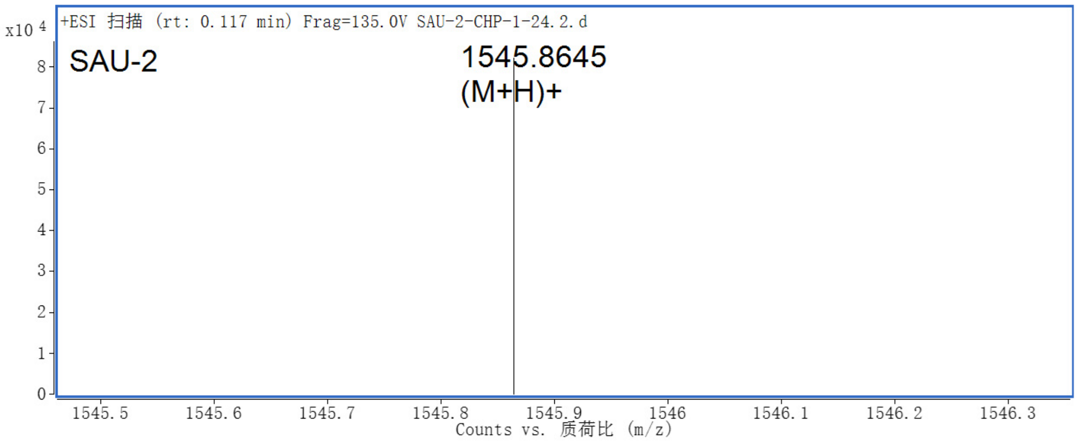

Figure S3. HR-MS for compound **SAU-2**

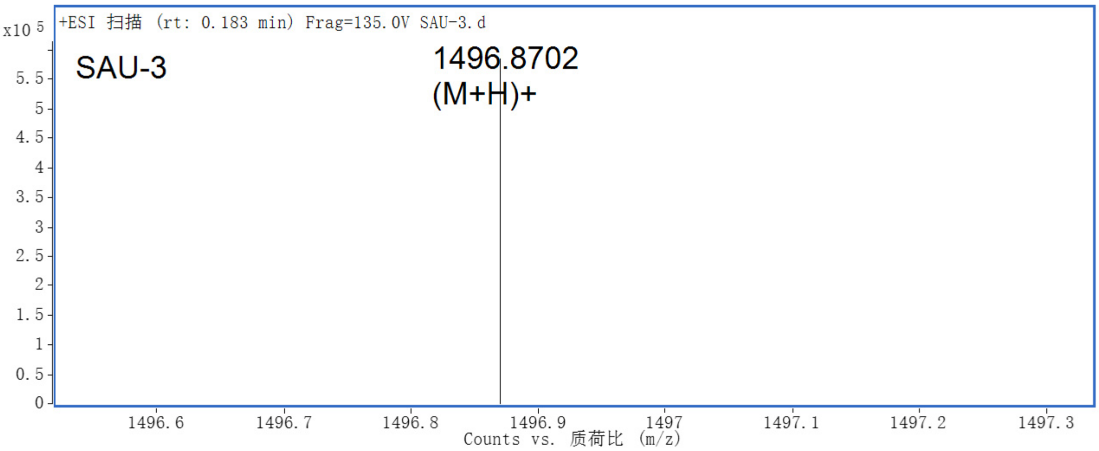

Figure S4. HR-MS for compound **SAU-3**

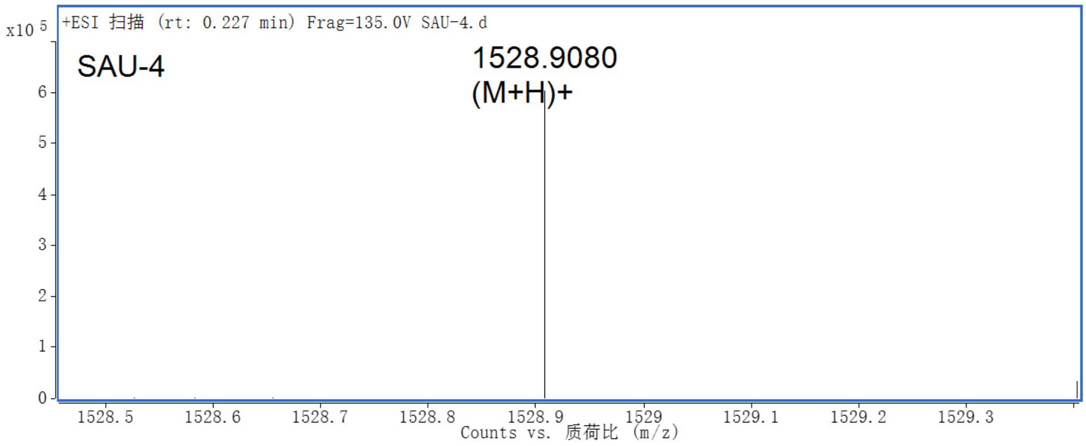

Figure S5. HR-MS for compound **SAU-4**

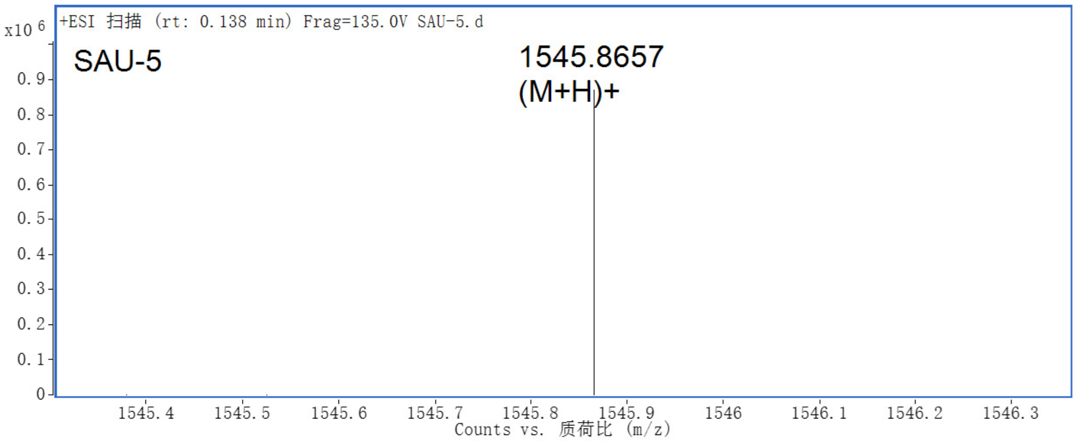

Figure S6. HR-MS for compound **SAU-5**

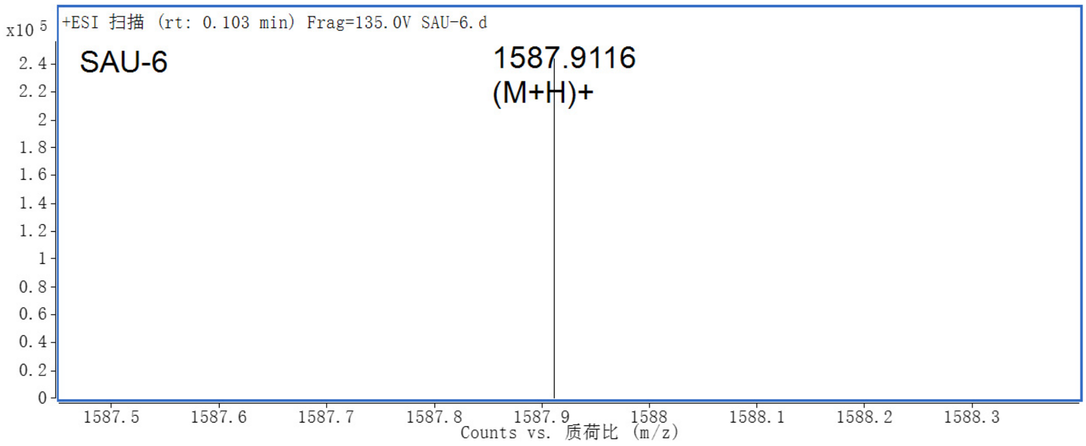

Figure S7. HR-MS for compound **SAU-6**

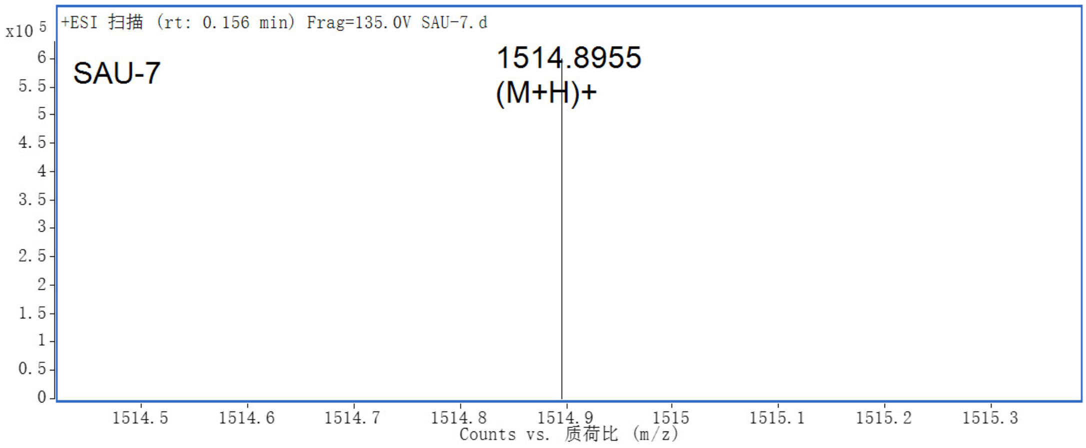

Figure S8. HR-MS for compound **SAU-7**

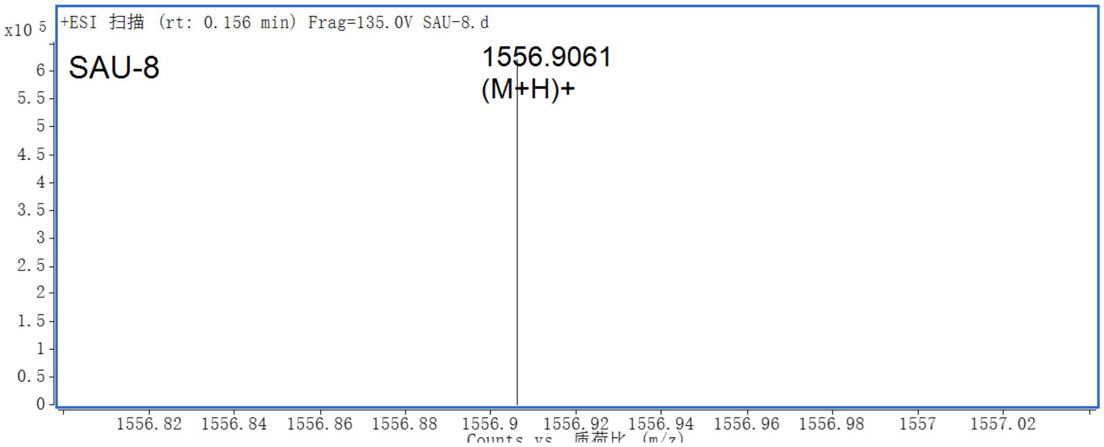

Figure S9. HR-MS for compound **SAU-8**

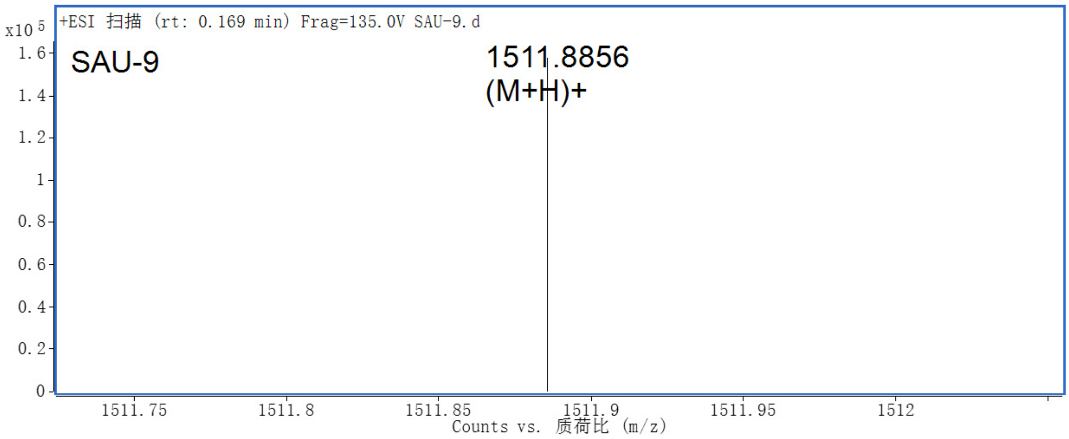

Figure S10. HR-MS for compound **SAU-9**

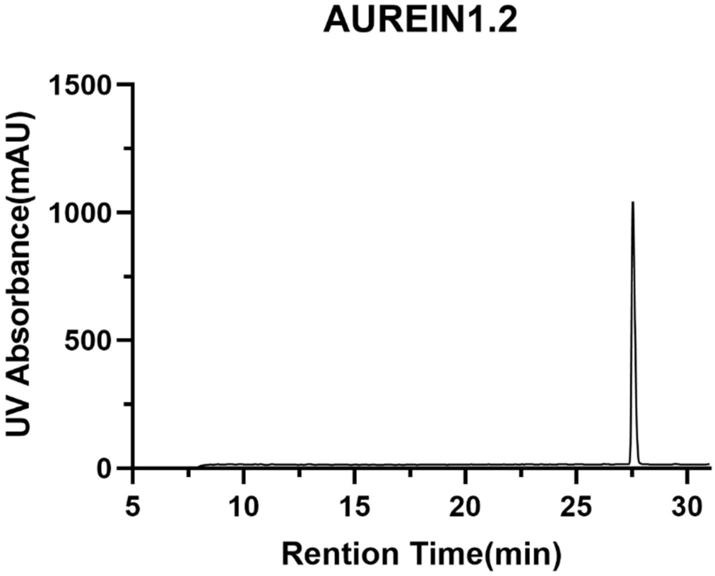

Figure S11. Analytical HPLC trace for compound **Aurein1.2**

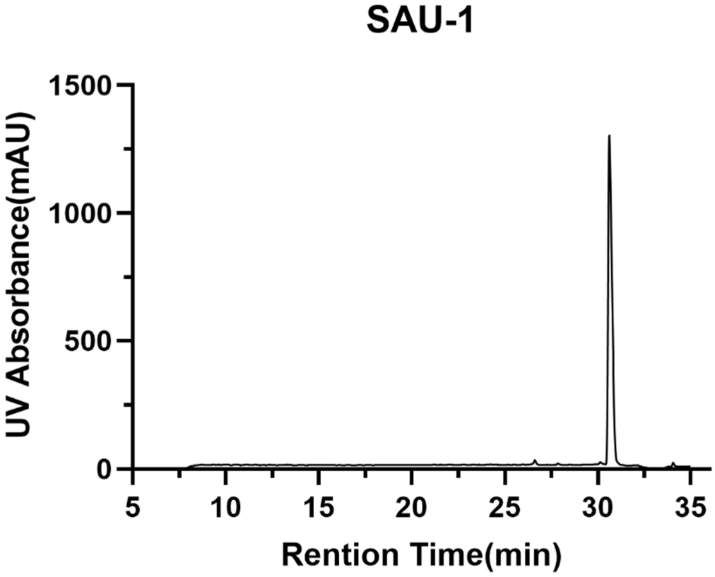

Figure S12. Analytical HPLC trace for compound **SAU-1**

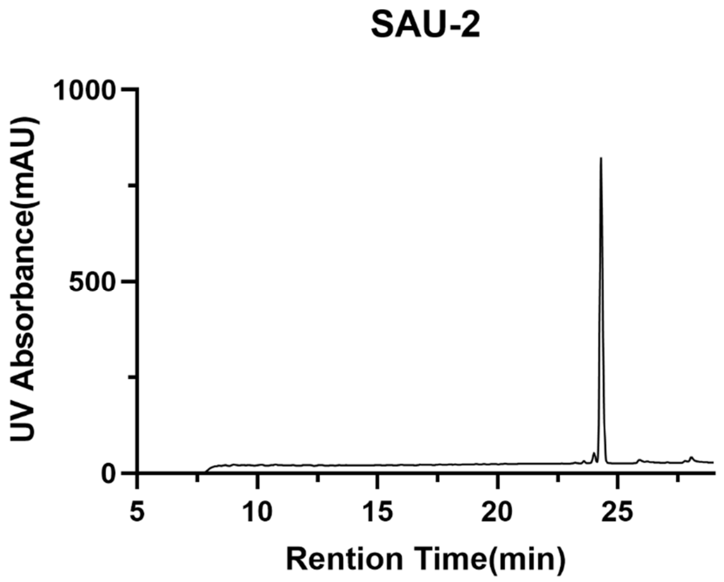

Figure S13. Analytical HPLC trace for compound **SAU-2**

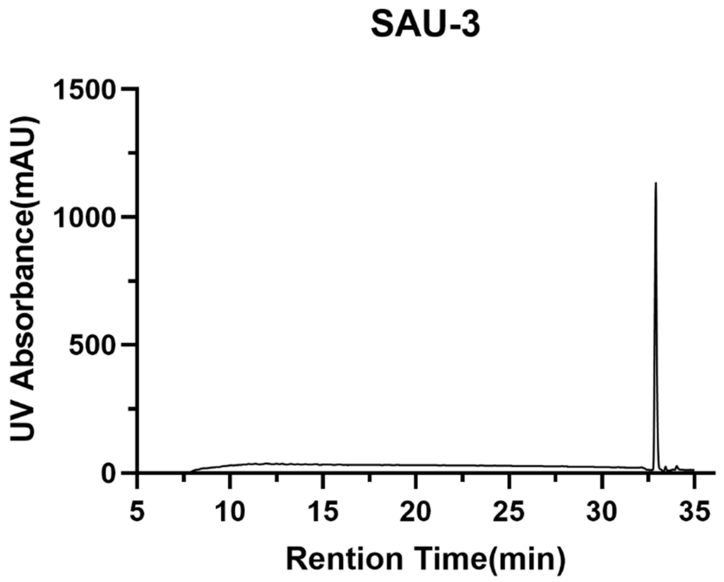

Figure S14. Analytical HPLC trace for compound **SAU-3**

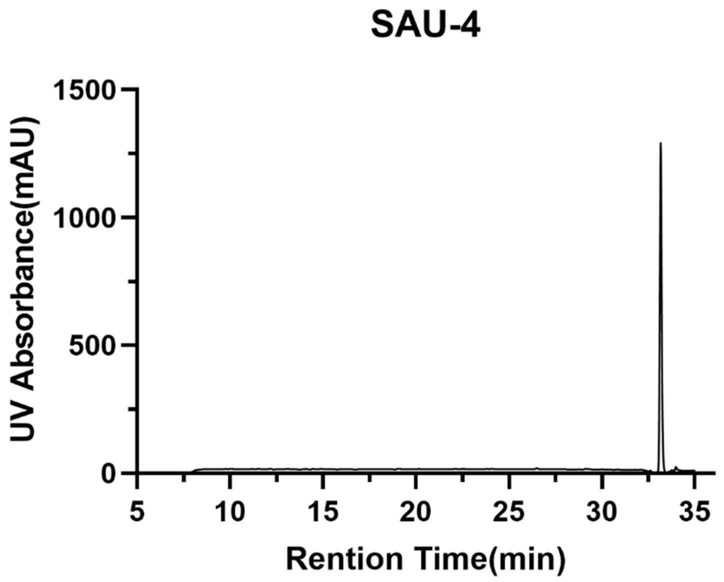

Figure S15. Analytical HPLC trace for compound **SAU-4**

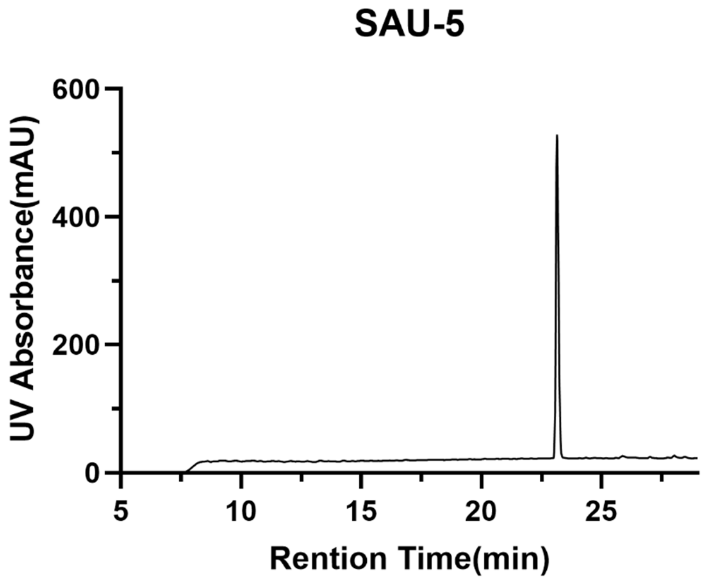

Figure S16. Analytical HPLC trace for compound **SAU-5**

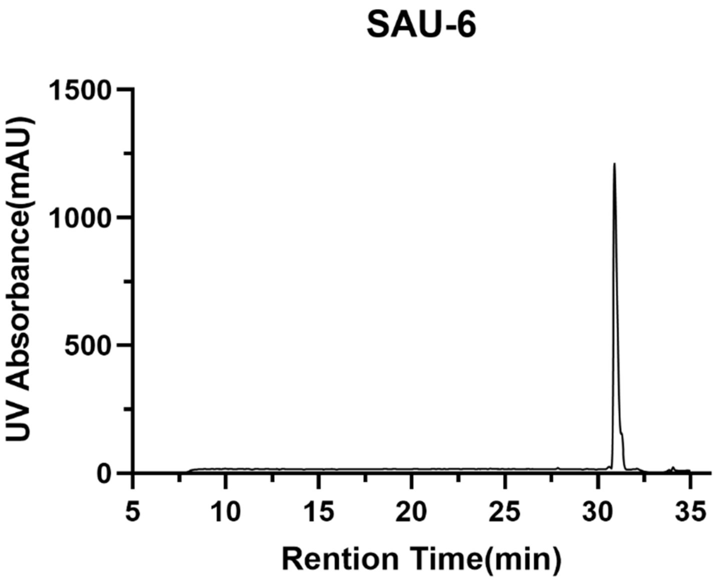

Figure S17. Analytical HPLC trace for compound **SAU-6**

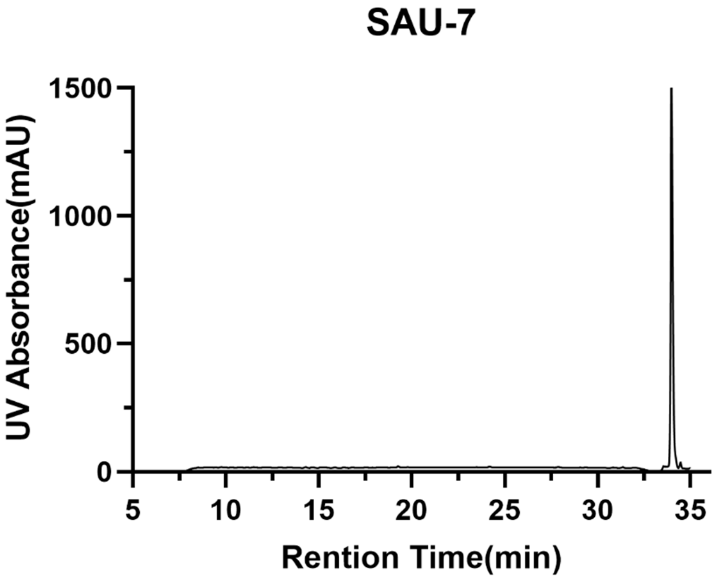

Figure S18. Analytical HPLC trace for compound **SAU-7**

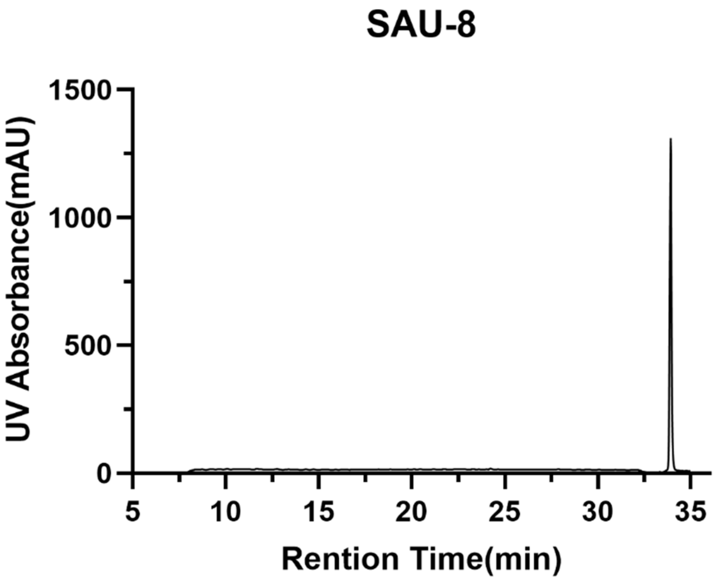

Figure S19. Analytical HPLC trace for compound **SAU-8**

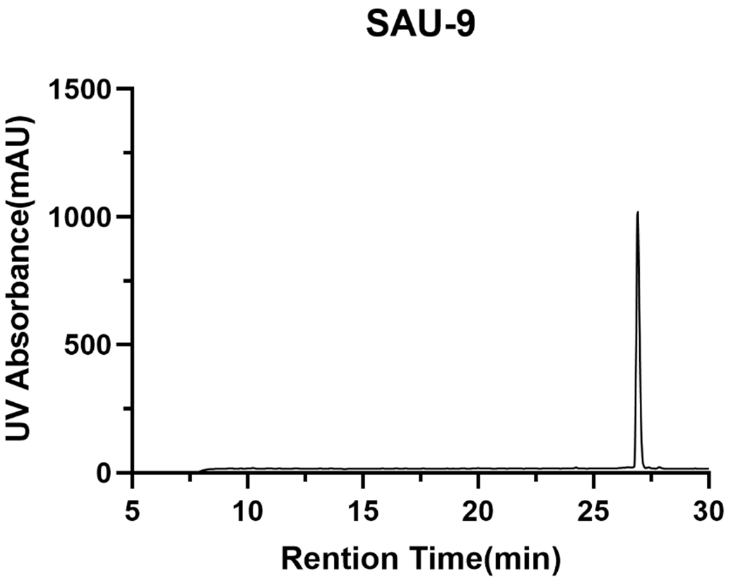

Figure S20. Analytical HPLC trace for compound **SAU-9**

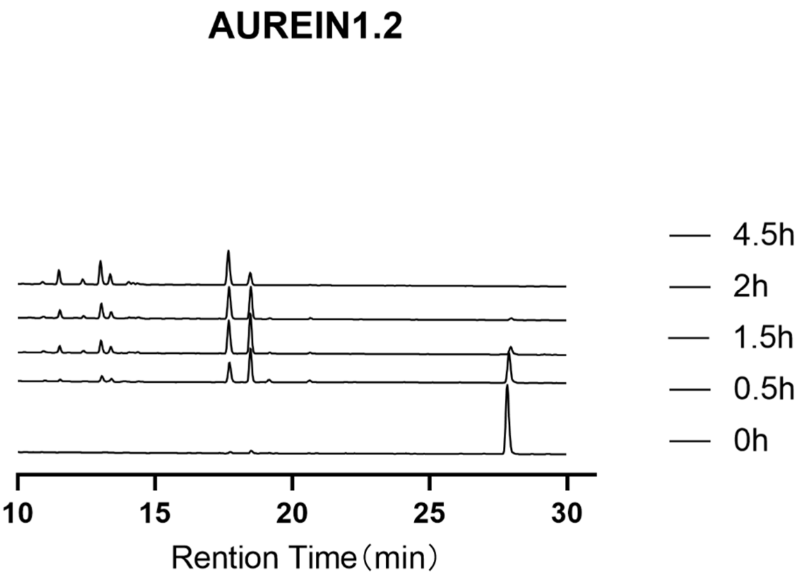

Figure S21. Analytical HPLC trace for Residual content of compound Aurein1.2

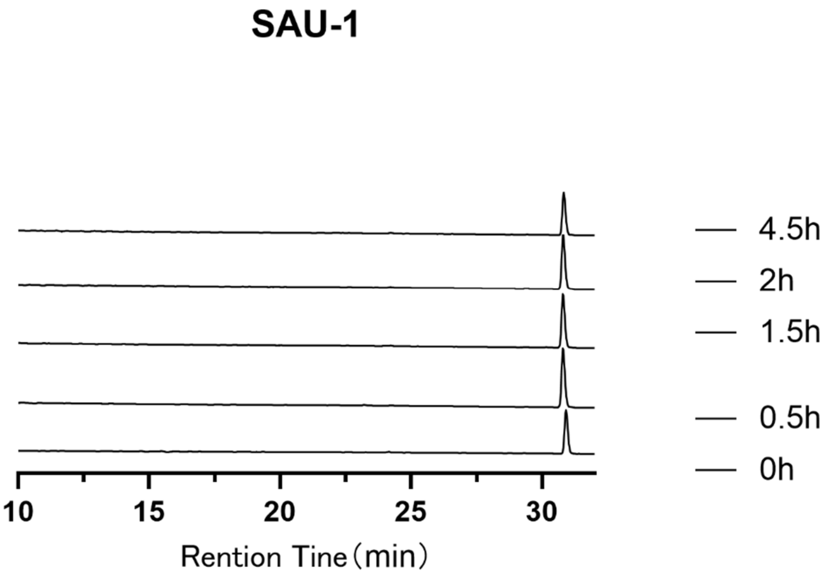

Figure S22. Analytical HPLC trace for Residual content of compound SAU-1

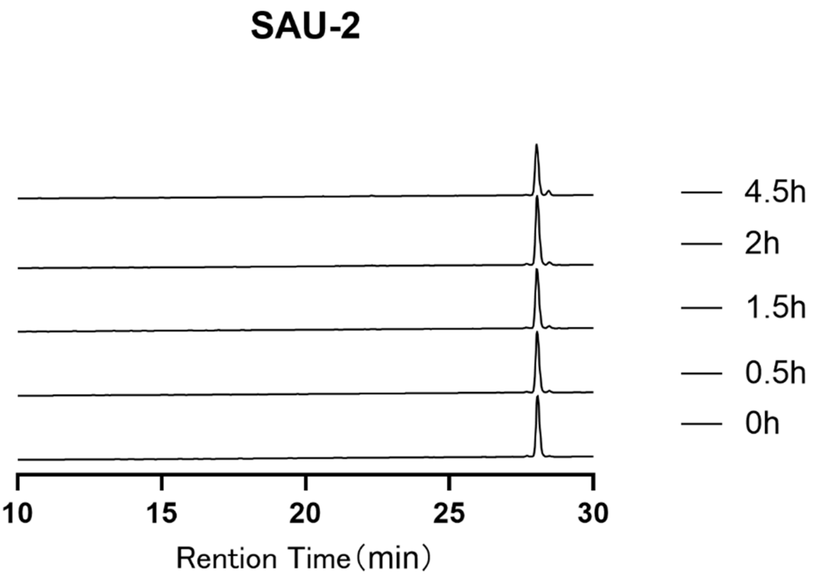

Figure S22. Analytical HPLC trace for Residual content of compound SAU-2

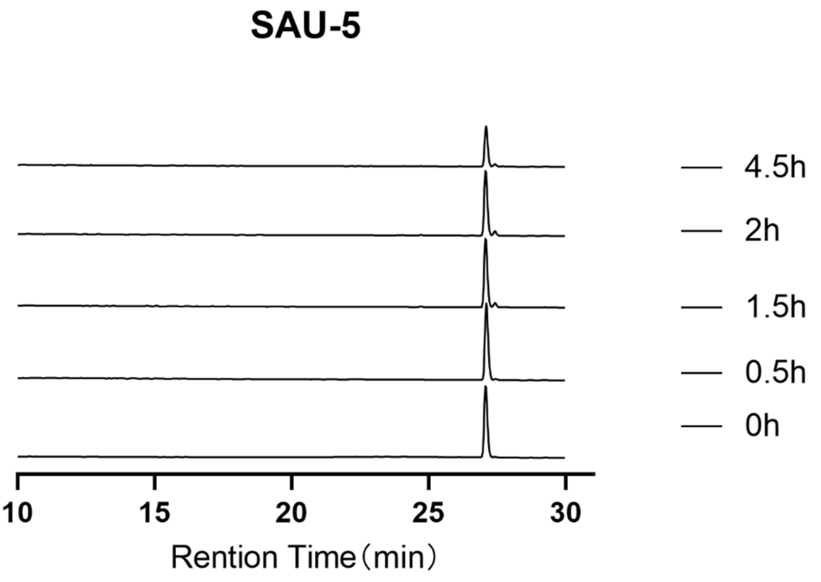

Figure S23. Analytical HPLC trace for Residual content of compound SAU-5

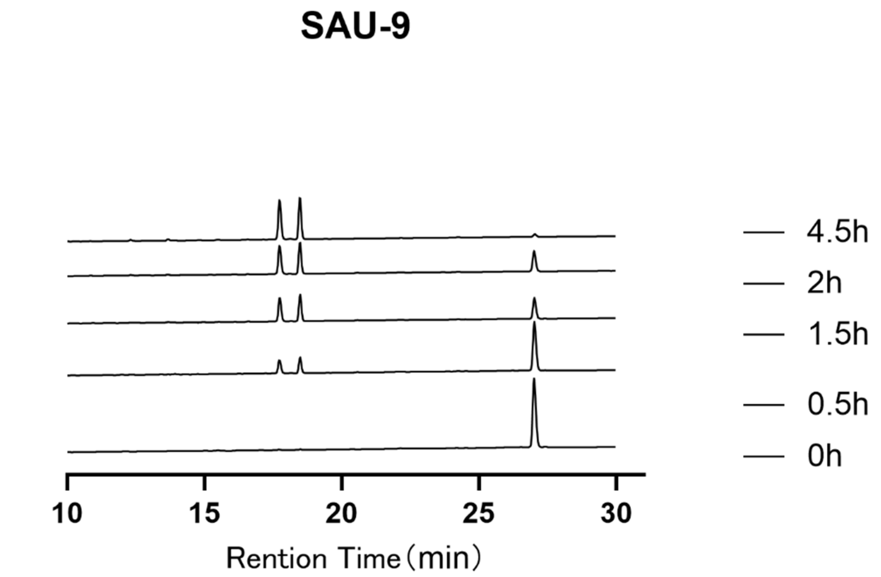

Figure S24. Analytical HPLC trace for Residual content of compound SAU-9

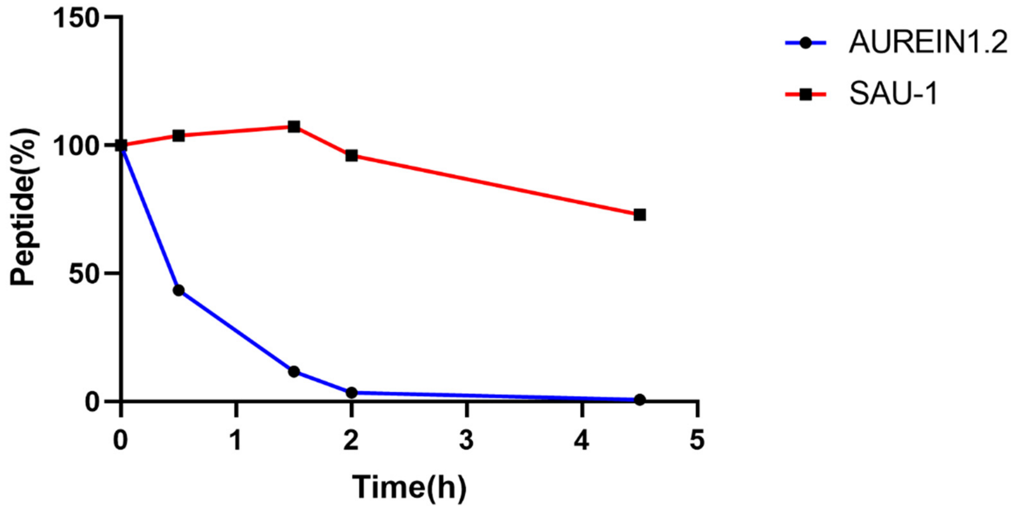

Figure S25. The hydrolytic stability of Aurein1.2 and SAU -1 was determined by high performance liquid chromatography

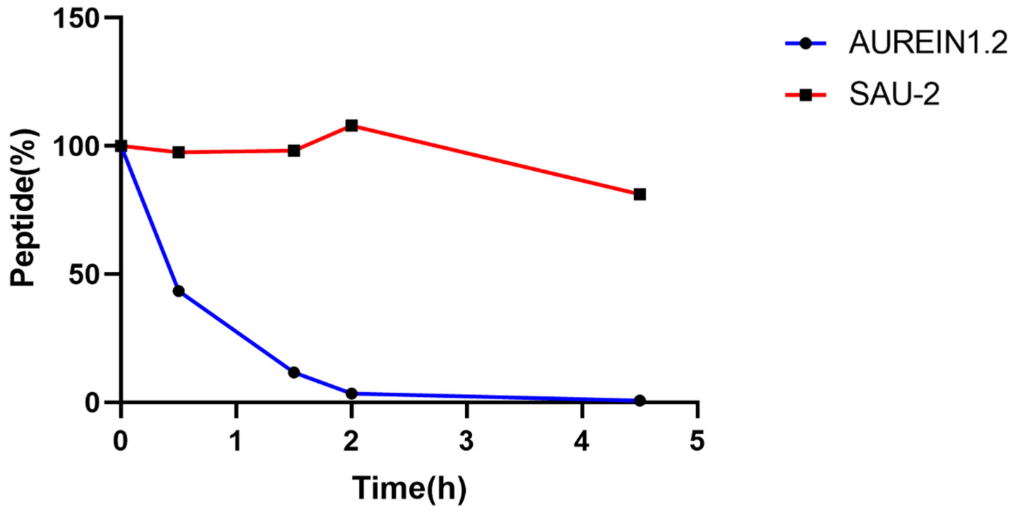

Figure S26. The hydrolytic stability of Aurein1.2 and SAU -2 was determined by high performance liquid chromatography

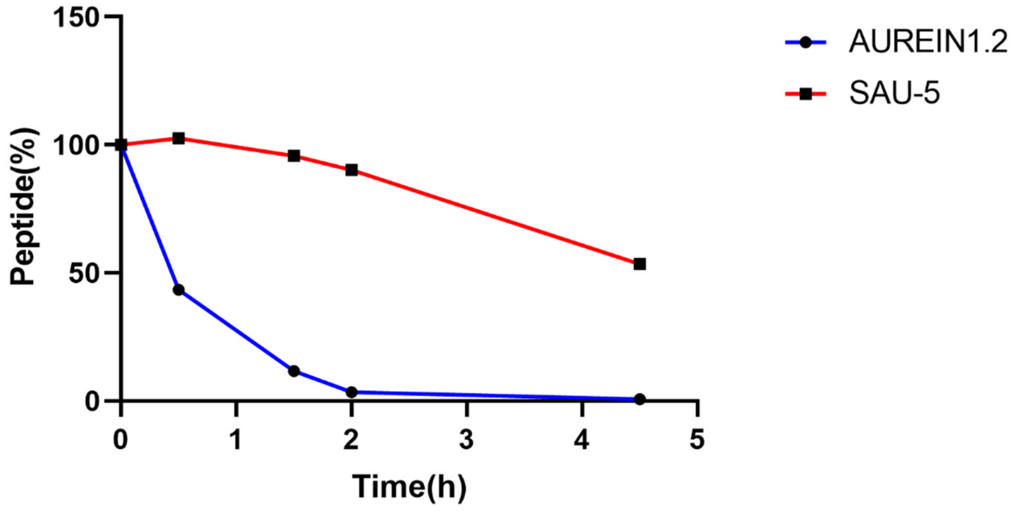

Figure S27. The hydrolytic stability of Aurein1.2 and SAU -5 was determined by high performance liquid chromatography

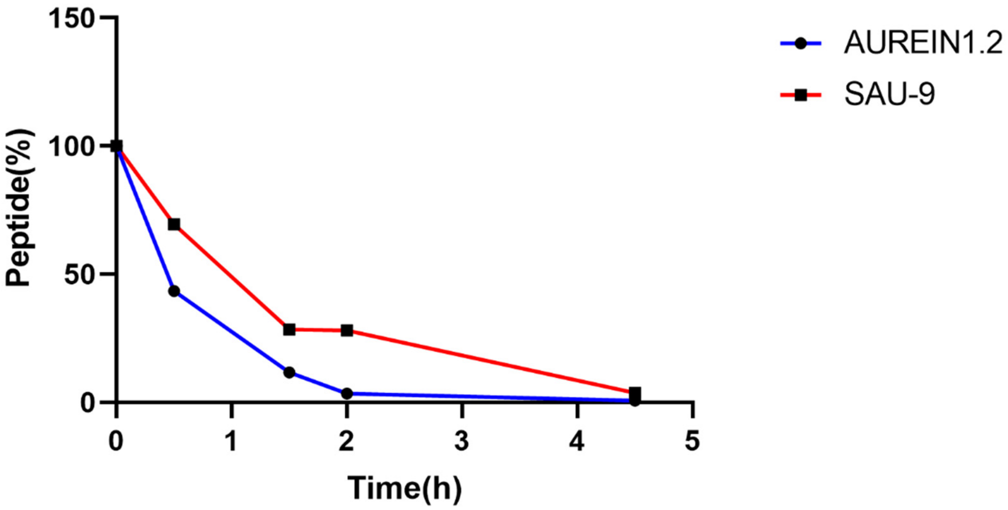

Figure S28. The hydrolytic stability of Aurein1.2 and SAU -9 was determined by high performance liquid chromatography
